# Accelerating cancer genomics research in Sub-Saharan Africa

**DOI:** 10.3389/fonc.2025.1531799

**Published:** 2025-05-29

**Authors:** Mahinè Ivanga, Berthe Amelie Iroungou, Stephane Bigoundou-Nzamba, Cyrille Bisseye

**Affiliations:** ^1^ Université des Sciences et Techniques de la Santé, Libreville City, Estuary, Gabon; ^2^ Registre des cancers – Institut de Cancérologie d’Akanda, Akanda, Estuary, Gabon; ^3^ Unité Mixte de Recherche Centre International de Recherche Médicale de Franceville, Service de Santé Militaire (UMR CIRMFSSM) – Hôpital d’Instruction des Armées Omar Bongo Odimba (HIAOBO), Libreville city, Estuary, Gabon; ^4^ Département d’Anatomie Cytologie Pathologiques et de Biologie des Tumeurs – Institut de Cancérologie d’Akanda, Akanda, Estuary, Gabon; ^5^ Unité de Recherche en Sciences Biologiques, Département de Biologie – Université des Sciences et Techniques de Masuku (USTM), Franceville, Gabon

**Keywords:** cancer, Sub-Sahara Africa (SSA), genomics, research, accelerating

## Abstract

Cancer is an increasing public health concern for Sub-Saharan African (SSA) countries, a region that is, unfortunately, already marked by the persistent presence of infectious diseases. Noticeably, in SSA, the universal approach to cancer treatment still prevails, whereas elsewhere, cancer treatment has shifted towards precision medicine. Definitely, the region faces many challenges that impede cancer genomics research despite its tremendous potential solution. Indeed, Genomics research could provide critical insights into the genetic determinants of cancers prevalent in SSA, enabling more precise and effective diagnosis, treatment, and prevention strategies tailored to local specificities. Therefore, shifting to precision medicine in this region is critical to tackle increase in cancer cases among SSA populations.

## Introduction

While the incidence and mortality rates of cancer are rising globally, significant disparities exist, particularly in sub-Saharan Africa (SSA) (1) where a complex interplay of genetic, environmental, and lifestyle factors contribute to variations in disease susceptibility and outcome. We are expecting the burden of cancer in the region to double, during the next two decades, due to population growth, ageing and lifestyle changes. This will, ultimately, result in 1.5 million new cases and 1 million deaths by 2040 (2). Despite the growing prevalence of cancer in sub-Saharan Africa (SSA), the majority of studies examining this disease in the region are still focused on populations from North Africa and South Africa (3). Nevertheless, cancer remains a significantly understudied field in SSA, particularly in the Central African region.

The approach to cancer treatment has shifted towards precision medicine, with genomics forming a pivotal foundation (4). The application of cancer genomics has the potential to enhance a number of aspects of cancer management, including prevention, early detection, prognosis prediction and the selection of appropriate treatment (5). To date, a multitude of precision oncology initiatives are primarily situated in Western countries with populations of European origin (6–9). However, it should be noted that European ancestry contributes only a subset of the human genetic variants and does not systematically characterise risk variants from other ethnic groups, such as those of African descent (10).

Recent studies have demonstrated the dearth of cancer genomics research on African populations and by Africans (3, 11). Indeed, the SSA region faces numerous challenges that restrict the potential of cancer genomics research across the continent. Nevertheless, it is imperative that cancer genomics becomes a priority in order to accurately define and assess the genetic determinants of cancer in populations of SSA. It is therefore evident that the region would derive significant benefit from the advent of cancer research breakthroughs and the implementation of personalised treatments. This paper considers the potential for accelerating cancer genomics research in SSA.

## Population-based cancer registries: understanding the landscape

Population-based cancer registries (PBCR) represent the principal source of information, as they are instrumental in monitoring the incidence, treatment, and outcomes of cancer cases, and are crucial for directing research and cancer control initiatives (12). Nevertheless, in numerous countries within the SSA, population-based cancer registries (PBCR) are either absent or inadequately maintained (13). Firstly, there is a tendency for cancer cases to be under-diagnosed and under-reported, which can be attributed to a lack of infrastructure, broken laboratory equipment, or a shortage of reagents that are necessary for cancer diagnosis. Secondly, the quality of the data is occasionally questionable due to inconsistencies in the reporting process, incomplete data records and a lack of technical expertise in data management. Furthermore, the absence of standardised definitions and classifications of cancer types can result in discrepancies in data reporting and hinder comparisons across regions. The absence of comprehensive data on cancer incidence and outcomes presents a significant challenge to research efforts and hinders the development of effective public health strategies.

It is evident that collaboration with international or regional partners is instrumental in guaranteeing the quality of the data produced and in developing standardised disease registry protocols. These partners provide training, guidance on best practice and tools for effective data collection. To illustrate, the African Cancer Registry Network (AFCRN) operates as the regional focal point for the International Agency for Research on Cancer (IARC) in coordinating cancer registration in SSA. It offers expert assessment of current challenges and technical assistance to address identified obstacles (13). The number of PBCR in SSA that can meet defined criteria of quality, including progressively complete population coverage increased from 21 in 2013 to 35 (in 25 different countries) by the end of 2021 (14). Nevertheless, in order to optimise PBCR in SSA, it is essential to ensure the effective organisation and support of cancer data collection from specialty data providers, accompanied by the provision of stable funding (15). Furthermore, the transition towards an electronic records management system should enable the overcoming of problems related to paper-based medical records and facilitate the clinical data sharing and linking to associated biological sources.

## Biobanks: a foundation for research

Biobanks are of vital importance for the storage of human biological samples, including DNA and tissue samples, which can be utilised in cancer genomic research and in clinical diagnosis. It is regrettable that a considerable number of countries in the SSA region are lacking in both organised and standardised biobanking systems, as well as the requisite financial resources to establish and maintain the necessary facilities. The collection of samples is frequently conducted in an *ad hoc* manner and the conservation of the samples is carried out in unsuitable conditions. This results in inconsistencies that compromise the validity of the research. It is therefore imperative that biobanking is conducted by local personnel with the requisite skills in order to facilitate the development of cancer genomics research in SSA. This will necessitate the deployment of human resources and the utilisation of global partnerships. Furthermore, ethical challenges associated with the establishment and utilisation of biobanks, in addition to the complexities surrounding data storage, exportation, utilisation and sharing, present significant obstacles to the creation of biobanks. Furthermore, the absence of guidelines that align with international standards can also engender patient distrust, as well as cultural perceptions and misunderstandings regarding the utilisation of biological samples.

In Africa, the blueprint model of biorepository operations and managements is the network of three biorepositories (located in West, South and East) established by the Human Heredity and Health in Africa (H3Africa) consortium to support biomedical research in the continent (16). It is recommended that SSA countries give priority to forming partnerships that can provide both financial and technical support, as well as developing centralised biobanks that adhere to international standards for sample collection and preservation. It is notable that no such biorepositories are present in Central Africa. In brief, the creation of research infrastructures comprising regularly linked biobanks and PBCRs represents a fundamental step towards the advancement of translational cancer research, encompassing the fields of aetiology, pathogenesis and prognosis. Furthermore, the establishment of cohorts with associated biospecimens will provide a reliable foundation for studies investigating risk factors and novel biomarkers for cancer in our populations (17). In terms of ethical considerations, it would be beneficial for SSA to work towards adapting existing clear ethical guidelines and regulatory frameworks to govern biobanking. This would help to strengthen local scientists, ensure transparency and foster public trust (18–20).

## Technical platforms: bridging gaps in technology

The incorporation of sophisticated technologies and platforms is imperative for advancing cancer genomics research. However, a substantial number of countries in the SSA region are lacking the requisite technological infrastructure to conduct advanced cancer genomics research, including the necessary high-throughput sequencing capabilities and the requisite analysis expertise (21, 22). Notably, there is a significant deficit in the number of trained professionals in the fields of genomics, bioinformatics, and data analysis in some countries in the SSA region. The primary reasons for the deficit in technology and the skilled workforce shortage are the insufficient funding allocated towards genomic research. Furthermore, there is frequently a dearth of collaborative networks among researchers, institutions, and international organizations, which are crucial for the exchange of knowledge, resources, and expertise. These limitations impede the effective utilisation of genomic data for cancer research, contributing to the perpetuation of knowledge gaps.

It is noteworthy that an increasing number of initiatives are encouraging a contemporary research approach by African investigators, thereby stimulating the study of the genomic basis of common diseases such as cancer. A number of ongoing Genome Wide Association Studies (GWAS) across Africa are seeking to identify genetic variants associated with cancer. These are being conducted by various research groups and consortia, including Evolving Risk Factors for Cancers in African Populations (ERICA), African Female Breast Cancer Epidemiology (AFBRECANE), Africa Esophageal Cancer Consortium (AfrECC) (23), and the Inclusive Cancer Care Research Equity (iCCaRE) for Black Men – Prostate Cancer Transatlantic consortium (CaPTC). The pan-African bioinformatics network for H3Africa (H3AfricaBioNet) has played a pivotal role in the advent of genomics research and the development of bioinformatics in the African continent. It has facilitated the establishment of essential infrastructure and trained a substantial cohort of researchers with analytical expertise (24). The network comprises over 30 nodes distributed across 15 African countries (25). Additionally, initiatives have been established with the objective of developing the capacity of the workforce in genomic research and bioinformatics, such as the African Society for Bioinformatics and Computational Biology. The provision of training in bioinformatics has now been extended to institutions in Eastern, Northern, Southern and Western Africa (24). Two principal organisations are spearheading the initiative to enhance the capacity of African researchers to conduct genomics research within their respective countries. These are the African Organisation for Research and Training in Cancer (AORTIC) and the Union for International Cancer Control (UICC) (26). It is evident that international collaborative initiatives with established research institutions can facilitate the transfer of knowledge. An exemplar of this is the public-private partnership between the Ugandan Cancer Institute and the Fred Hutch Cancer Center (Seattle, WA, USA) (27). The scope of this partnership covered research, training, and biomedical laboratories. Similarly, Syndicate Bio recently partnered with the Nigeria’s National Institute for Cancer Research and Treatment (NICRAT) to launch the Cancer Genome Nigeria Project. In addition, local establishments can enable to leverage modern technologies effectively. For example, the partnership between the Nigerian Sovereign Investment Authority and Lagos University Teaching Hospital (Lagos, Nigeria) enabled to increase Lagos University Teaching Hospital (LUTH) radiotherapy capacities (28).

## Moving away from one-size-fits-all

Given the highly diverse nature of cancer, SSA has to move from the one-size-fits-to-all conventional approach, consisting of a single treatment protocol based on the average response within a population (mostly non-African), towards more personalised treatments based on patient characteristics and mutations. Patients with cancer are often diagnosed at an advanced stage in SSA. The field of cancer genomics offers unprecedented opportunities for understanding cancer biology and developing targeted treatments specific to African populations. For instance, recent studies have shown the high contribution of BRCA 1 and 2 genes mutations in breast cancers of African women (29). Indeed, pathogenic BRCA1/2 mutations and haplotypes unique to Nigerian that could be used to prevention, early detection and personalised treatment planning in Nigeria have been identified (30). Besides, the first large-scale African genomic study to date has identified three loci associated with increased risk of prostate cancer and that are unique to African populations (31). Altogether, these studies highlight the need for population-specific genetic screening. If GWAS have identified several thousand locations of genes related to cancer in non-African populations, the risk in Sub-Saharan people is not clearly known. We note that the Men of African Descent and Carcinoma of the Prostate (MADCaP) network has developed a genotyping array specifically designed to Afrocentric variants and to explore polygenic risk scores (32). Large scale studies with high throughput techniques are required to characterise candidate genes for African-based cancers (22) and growing GWAS are currently ongoing through different consortia, across SSA (23). Accordingly, the implementation of low-cost genetic testing covering relevant pathogenic variants and genetic counselling services in African healthcare systems should improve cancer prevention strategies, early diagnosis, and choices for therapeutic. However, very few African countries are able to integrate genetic services in their healthcare systems due to different level of advances in genomics. A path would be to develop a network of at least four genomic hubs (located in West, East, Central and South Africa) with high-throughput sequencing capabilities to address regional cancer health challenges.

In SSA, investing in cancer genomics often takes a backseat to other pressing health issues, such as infectious diseases, which can lead to underfunding and inadequate policy support. Clearly, the COVID crisis made the policy makers aware of the importance of genomics in healthcare. Thus, a better understanding of cancer genomics is a key to refine healthcare policies and local resource allocation. While, regional collaborations among stakeholders should be heightened to ensure the financial viability and the full benefits of such a scientific initiative. In addition, the local lack-of-knowledge on the subject remains a key barrier which needs to be addressed. As in cancer screenings carried out in some African countries (33), sensibilisation programs involving influential community leaders is essential to increasing participation rates to research studies and adherence to early prevention. Developing culturally tailored health education programs that connect genomic concepts to familiar ideas like place of origin and ancestry transmission should make the information more relatable and impactful for local populations. Lastly, SSA countries should set-up national guidelines for genomic medicine and cancer genetics, implement ethical review boards to monitor genomic research and to ensure better cancer outcomes. There is emerging interest about the extent to which Ubuntu, a South African communitarian ethic and theory of justice, could offer guidance for the equitable governance of genomics in Africa. It is noteworthy that Ubuntu can be resumed by seven principles: solidarity, reciprocity, inclusivity, open sharing, deliberative and consensus decision-making, mutual trust, and accountability (34).

## Conclusion

In summary, by addressing challenges related to cancer registries, biobanks, and technological platforms, SSA can create a robust framework for cancer research. Through collaborative efforts, investment, and policy reform, the region can harness its genetic diversity to foster innovations in cancer prevention and treatment tailored to its unique population. Strategies should promote local knowledge, experiences, and solutions. The future for cancer genomics research is to grow large studies across the major regions and populations of SSA.

## Data Availability

The original contributions presented in the study are included in the article/Supplementary Material. Further inquiries can be directed to the corresponding author.
